# A 5-Year Suicide Rate of Adolescents Who Enrolled to an Open Dialogue-Based Services: A Nationwide Longitudinal Register-Based Comparison

**DOI:** 10.1007/s10597-023-01106-0

**Published:** 2023-03-14

**Authors:** Tomi Bergström, Jaakko Seikkula, Selma Gaily-Luoma, Jouko Miettunen, Mia Kurtti

**Affiliations:** 1 Department of Psychology, University of Jyväskylä, Jyvaskyla, Finland; 2 Department of Psychiatry, Wellbeing service county of Lapland, Kemi, Finland; 3 Faculty of Health and Sport, University of Agder, Kristiansand, Norway; 4 Center for Life Course Health Research, University of Oulu, Oulu, Finland; 5 Medical Research Center Oulu, Oulu University Hospital and University of Oulu, Oulu, Finland; 6 Faculty of Social Sciences, University of Helsinki, Helsinki, Finland

**Keywords:** Child and adolescent psychiatry, Family therapy, Mortality, Need-adapted approach, Mental health services

## Abstract

In the Open Dialogue (OD) based psychiatric services adolescent patients receive less medication and are more often treated within an outpatient setting as compared to standard services. An evaluation of the possible risks of implementing OD are required. The aim of this longitudinal register-based study was to evaluate how treatment under OD is associated with the probability of suicide as compared standard psychiatric care. Study included all 13- to 20-year-old adolescents who enrolled to a psychiatric service in Finland in 2003–2013. The OD-group included adolescents whose treatment commenced in the Western Lapland area (*n =* 2107), this being the only region where OD covered all psychiatric services. The comparison group (CG) included rest of Finland (*n =* 121,658). Information was gathered from onset of treatment to the end of the 5-year follow-up or death. In a multivariate Cox regression there were no statistically significant differences in 5-year suicide hazard ratios between OD and CG.

## Introduction

Open Dialogue (OD) is a social network-oriented model of service delivery in mental healthcare originated in the Finnish catchment area of Western Lapland (Seikkula et al., 2011). OD includes both the structural principles describing ways of (re-)structuring entire mental health services and therapeutic principles outlining the specific treatment approach (Table 1) (von Peter et al., 2019). The aim of both is to guarantee an immediate need-adapted and dialogical response to mental health crises regardless of diagnosis. In OD-based services all relevant people are gathered in joint network treatment meetings as soon as possible to create a shared understanding of mental health crises within reciprocal dialogues (Bergström et al., 2022a; Seikkula et al., 2011). Thereafter those service team members who participate in the first meeting are responsible for ensuring the needs adaptedness and psychological continuity of treatment over the entire treatment process (Bergström et al., 2022b) (Table 1).

**Table 1:** The organizational and therapeutic principles of Open Dialogue approach

Structural principles	Key elements of fidelity to therapeutic approach of OD
Immediate help	Two (or more therapists)
Social network perspective	Participation of family and/or network
Flexibility and mobility	Use of open-ended questions
Psychological continuity	Responding to clients’ utterances
Therapeutic principles	Emphasizing the present moment
Tolerance of uncertainty	Eliciting multiple viewpoints
Dialogue	Use of a relational focus in the dialogue
	Responding to problematic discourse in a matter-of-fact style
	Emphasizing the clients’ own words, not symptoms
	Conversation among professionals within meetings (reflection)
	Being transparent
	Tolerating uncertainty

In register-based cohort studies OD has demonstrated good clinical and functional long-term outcomes in the treatment of young people with mental health difficulties (Bergström et al., 2018, 2022a; Buus et al., 2019). In Western Lapland OD-based treatment has also been associated with a lower all-cause standardized mortality ratio (SMR) in the treatment of first-episode psychosis as compared to standard care, but no significant differences in suicide-mortality have been found between the two treatment models (Bergström et al., 2018). In 2010’s, the Western Lapland catchment area has demonstrated higher annual population proportions of suicides as compared to many other parts of Finland (Partonen et al., 2020, 2022; THL, 2022).

In comparison to standard psychiatric services, patients treated in the OD model less often receive medication (Bergström et al., 2022c) and diagnoses (Bergström et al., 2022a), and are more often treated within an outpatient setting even during serious mental health crises (Bergström et al., 2018; Seikkula et al., 2011). An evaluation of the possible risks of implementing OD-based approaches requires an examination of OD’s association to suicide risk.

The aim of this nationwide longitudinal register-based cohort study was to analyse the 5-year suicide rate and suicide risk factors among the cohort of all adolescents who enrolled to psychiatric services in Finland between 2003 and 2013. The more specific aim was to examine whether service type (OD-based services vs. standard mental health care) was associated with differences in 5-year suicide rate among this population of adolescents receiving mental health services.

## Methods

### Study Design

Finland is a Nordic country with a population of 5 million. Mental health services in Finland are publicly funded, and the municipalities are responsible for providing services for all their residents. Adolescents who need psychiatric care are usually referred from primary and/or school healthcare services to a secondary healthcare system, operating under the 21 Finnish hospital districts funded by a consortium of municipalities.

The research cohort included all adolescents aged 13–20 who received psychiatric treatment in Finland during the period of January 1, 2003 to December 31, 2013 (N = 123,765). The study materials were gathered from the nationwide social and health care registers by the Finnish social and health data permit authority Findata in the years 2020 and 2021. All available entries (prior and after onset of adolescent treatment) were gathered from registers up to the end of the year 2018, enabling continuous follow-up for the entire cohort until death or the end of the 5-year follow-up, whichever occurred first.

The Open Dialogue-group (OD) included all people who received adolescent psychiatric treatment in the catchment area of Western Lapland during the study period. The hospital district of the catchment area covers the South-Western parts of Finnish Lapland, with a population of approx. 65,000 in years 2003–2013. Open Dialogue based services have been systematically developed in the catchment area since the 1980’s, and at the study period, it was the only region in Finland where all adolescent psychiatric services were based on OD, regardless of diagnosis (Bergström et al., 2022a; Seikkula et al., 2011). The comparison group included all adolescents whose treatment was commenced outside of the Western Lapland region.

According to the fidelity criteria of OD (Olson et al., 2014), the approach has two fundamental features: (I) it is a community-based, integrated treatment system that makes an effort to engage families and social networks from the very start of the treatment and (II) a dialogical practice/distinct form of therapeutic conversation takes place within the network treatment meetings. Hence, OD-based services differ from more conventional medical model, in which care is usually organized at the level of the individual, and is based predominantly on the diagnosis and immediate symptom reduction in line with predetermined group-level and diagnosis-specific treatment guidelines (Razzaque & Stockmann, 2016). Note however, that as there are variation in how mental health care services and treatment is actually arranged in different parts of Finland, comparison group of this study represent standard care only at a general level.

More detailed information on dialogical practice (Seikkula et al., 2011; Olson et al., 2014) and OD in Western Lapland adolescent psychiatric services, including 10-year treatment outcomes of first-onset adolescent patients, are presented elsewhere (Bergström et al., 2022a).

### Variables

For each case the service entry time was set to be a first register-entry in adolescent services. End of the follow-up was set to be 1825 days after first entry or death, whichever occurred first. Variables were formed by combining information from different registers.

Information on overall and cause-specific mortality for the entire sample were obtained from registers of Statistics Finland. The outcome of interest was suicide-mortality during the 5-year follow-up from the first register entry of adolescent psychiatric treatment.

### Statistical Methods

At the first step of the statistical analysis, T-test and chi-square tests were used to compare group differences. Population proportions (n of suicides per inclusion year, divided by the population of a similar age in the catchment area) were used to compare the average 5-year suicide rates per inclusion year between OD and CG. To calculate standardized mortality ratios (SMR), Finnish public registers provided by Statistic Finland were used to create age- and sex-specific estimations of expected all-cause and suicide deaths in 5-year-follow-up.

At the second step of the analysis, a stepwise Cox regression model was used to examine risk factors for suicide in the entire sample during the 5-year follow-up. The studied factors are presented in Table 2. Prior analysis proportional hazard assumptions were checked and confirmed for all variables. Stepwise (conditional) method was used to identify those factors whose inclusion gives the most statistically significant improvement of the fit of the proportional hazards model on probability of suicide during 5-year follow-up. To produce more detailed descriptive information, post-hoc survival analyses were performed with identified risk-factors. Since the main aim of the first two steps of the analyses was to produce descriptive information on 5-year suicide rate and suicide risk-factors among the cohort of all adolescents who enrolled to psychiatric services, the first two steps were not adjusted for multiple comparison.

**Table 2:** Potential risk factors of suicide

Factor	Description
Age	Age at onset in adolescent psychiatric services
Gender	Male/female
Substance abuse	Yes, if there was one or more register entries with substance use disorders (F10-F19) during the first year of service entry
Psychosis	Yes, if there was one or more register entries with psychosis (F20-F29) during the first year of service entry
Mood disorder	Yes, if there was one or more register entries with mood-disorder (F30-F39) during the first year of service entry
Anxiety disorder	Yes, if there was one or more register entries with anxiety-disorder (F40-F48) during the first year of service entry
Prior mental health treatment	Yes, if there was one or more visits in mental health services and/or psychiatric hospital and/or psychiatric medication purchases prior onset of adolescent psychiatric treatment
Prior placements outside of family	Yes, if there was one or more child protective placements outside of family prior onset of adolescent psychiatric treatment
Prior general hospital treatment	Yes, if there was one or more treatment contacts in general hospitals prior onset of adolescent psychiatric treatment
Treatment group (OD/CG)	Dummy-coded variable indicating whether or not adolescent psychiatric treatment was commenced in the Western Lapland catchment area (Open Dialogue, OD) or in other parts of Finland (Comparison group, CG)

At the third step of the analysis, we used a Cox regression to test the two-sided hypothesis that service type (OD-based services vs. standard mental health care) associates with a difference in 5-year suicide hazard ratio. First, the exposure variable (OD/CG) was added in a univariable model. At the next step a multivariable model was created by adding potential confounders into the model. Potential confounders were identified in a previous step of the data analysis by using the following definition (Babyak, 2009): a potential confounder was associated with the outcome (suicide-mortality) and the predictor of interest (OD/CG). The statistical significance level was set to be the two-tailed p < .05. Bonferroni correction was used to adjust for multiple comparison. The Adjusted hazard ratios (aHR) were used to estimate size and direction of the effect.

All analyses were conducted via IBM SPSS Statistics 27 for Windows.

## Results

### Group Characteristics

The average age at the service entry of adolescent psychiatric treatment was 16 years in OD and 15.8 in CG. Difference was statistically significant (p < .001). There were no significant differences in the gender-distribution between the two groups. Aligned with the main premise of OD, the OD-group had spent less in a psychiatric hospital during follow-up and received fewer dispensed antidepressants and antipsychotics (Table 3).

**Table 3:** Group characteristics

	Open dialogue (OD) *n* = 2107	Comparison group (CG) *n* = 121,658	Statistic (t-test or chi-square)	p
Demographics				
Age (M (SD))	16 (2.2)	15.8 (2.3)	− 4.2	< .01
Gender, female	1285 (61%)	72,382 (60%)	1.9	.17
Services prior^a^ onset in adolescent clinic				
Mental health treatment	607 (28%)	33,173 (27%)	2.5	.11
Treatment in general hospitals	1246 (59%)	63,638 (52%)	38.7	< .01
Placement outside of family	328 (16%)	23,885 (19%)	21.8	< .01
Clinical characteristics				
Any F-diagnosis at onset^b^	350 (17%)	87,850 (72%)	3126	< .01
Antidepressants at onset^b^	252 (12%)	36,053 (30%)	312	< .01
Antipsychotics at onset^b^	112 (5%)	21,183 (17%)	213	< .01
% in hospital in 5-year follow-up (M (SD))	0.4 (3.5)	1 (4.7)	6.3	< .01
Outcome				
Death	20 (0.9%)	1038 (0.9%)	0.225	.64
Suicide	6 (0.3%)	428 (0.3%)	0.266	.61

^a^Including also register entries prior inclusion period (year 2003)

^b^First treatment year

The annual incidence of adolescents’ mental health service users per 1000 people of a similar age was 28 in OD and 22 in CG. Even though the relative proportion of service users was higher in OD, there were significantly fewer service users with psychiatric diagnoses. As compared to the CG, the OD group showed smaller diagnosis proportions in the first treatment year as follows: for any F-diagnosis 17% vs. 72%, for substance abuse (F10–F19) 1% vs. 3%, for psychosis (F20–F29) 1% vs. 4%, for mood disorder (F30–F39) 6% vs. 30%, and for anxiety disorder (F40–48) 7% vs. 26%. Differences in diagnostic distributions are due to OD principles, in which diagnostic procedures are not regularly used or required for the arrangement of mental health services.

### Suicides and Suicide Risk-Factors

The average population proportion of suicides per inclusion year was 6.4/100,000 same aged people (95% CI 1.5–11.5) in OD-group and 7.6/100,000 (95% CI 7.1–8.1) in CG-group. All cause 5-year standardized mortality ratio in OD was 3.3 (95% CI 2.7–3.9) and in CG 3.4 (95% CI 3.3–3.5). SMR for suicides in OD was 3 (95% CI 1.8–4.2) and in CG 3.7 (95% CI 3.5–3.9).

In a total sample those who died by suicide were more likely males and demonstrated overall more severe psychiatric symptomatology at the service entry (Table 4). There were no differences in prior psychiatric treatment contact, general hospital visits prior to onset of treatment or placements outside of the family between those who died by suicide and those who didn’t. Aligned with potential confounding by indication, those who died by suicide had on average spent more time in a psychiatric hospital during follow-up (5% vs. 1% of total follow-up time) and had more likely received antidepressants (45% vs. 29%) and antipsychotics (37% vs. 17%) during their first treatment year.

**Table 4:** Clinical and demographic characteristics of those who died by suicide during 5-year follow-up

	Suicide N = 434	No suicide N = 123,332	Statistic (t-test or chi-square)	p
Demographics				
Age at onset in adolescent clinic (M (SD))	17 (2.1)	15.8 (2.2)	− 12	< .01
Gender, female	167 (38%)	73,500 (60%)	80	< .01
Services prior^a^ onset in adolescent clinic				
Mental health treatment	121 (28%)	33,659 (27%)	0.76	.783
Treatment in general hospitals	209 (48%)	64,675 (52%)	3.1	.075
Placement outside of family	96 (22%)	24,118 (20%)	1.8	.179
Clinical characteristics at the service entry^b^				
Substance abuse (F10–F19)	69 (16%)	3438 (3%)	270	< .01
Psychosis (F20–F29)	70 (16%)	5317 (4%)	145	< .01
Mood disorders (F30–F39)	190 (44%)	36,516 (30%)	41.7	< .01
Anxiety disorders (F40–F48)	116 (27%)	31,675 (26%)	0.25	.619
Any F-diagnosis	350 (81%)	87,850 (71%)	18.7	< .01

As Finnish social and health data permit authority doesn’t allow to report frequencies < 6, within group differences in OD are not reported in detail. According to within group-analysis of suicides under OD, there were no statistically significant differences with diagnosis, dispensed medications and hospital-admissions of those who died by suicide as compared to those who survived.

^a^Including also register entries prior inclusion period (year 2003)

^b^First treatment year

Identified suicide risk factors are presented in Table 5. The most notable risk-factor in the total sample was diagnosed substance abuse disorder during the first treatment year. In post hoc survival analyses one or more entries of an alcohol (age and gender adjusted hazard ratio (aHR): 3.4, 95% CI 2.4–5, p < .001), cannabis (aHR: 2.7, 95% CI 1.4–5.4, p < .001) or amphetamine/stimulant related (aHR: 4.5, 95% CI 2.4–8.6, p < .001) disorder increased the risk of suicide. Risk for suicide was notably high (aHR: 10, 95% CI 6–16, p < .001) for those who had diagnoses for polysubstance use (i.e. two or more different substances) as compared to those with only one or no substance-related diagnoses during first treatment year.

**Table 5:** Identified risk factors for suicide

Risk factor	Hazard ratio	95% CI	p
Gender, male	2.2	1.8–2.8	< .001
Age	1.2	1.1–1.3	< .001
Substance abuse disorder	3.7	2.8–4.9	< .001
Psychosis	2.3	1.8–3.1	< .001
Mood disorder	1.8	1.5–2.1	< .001

### Outcome

The association between service type (OD vs. standard care) and 5-year suicide hazard ratio (HR: 0.8, 95% CI 0.4–1.8, p: .6 > Bonferroni corrected p-value .025) was not statistically significant. After adding potential confounders (age, substance abuse, psychosis, mood disorder) into the model, the effect of service type remained statistically non-significant (aHR: 1.1, 95% CI 0.5–2.5, p: .8).

## Discussion

This nationwide register-based cohort study investigated adolescent suicide risk during a 5-year follow-up period after first entry into psychiatric services, comparing Open Dialogue-based services and standard psychiatric treatment. No significant difference in suicide mortality between Open Dialogue-based treatment and standard psychiatric treatment was found. In both services the standardized mortality ratio of an adolescent mental health service users’ risk of suicide was over three times higher than that in the general population of similar age. The probability of death by suicide during the 5-year period was significantly higher for males and those with more severe psychiatric symptomatology. From all identified risk-factors in the entire sample, the most notable was the diagnosis of a substance abuse at the beginning of treatment. However, due to the observational nature of study, we were not able to determine the causality of any observed associations.

Our main findings are in line with previous studies on suicide risk-factors of young people with mental health difficulties (Bilsen, 2018; Lahti et al., 2014). However, results are partially contradicting with Finnish national social and healthcare indicators, in which the annual population proportion of suicides in Western Lapland catchment area has been higher than in most other parts of Finland (Partonen et al., 2022). Earlier longitudinal studies with other age- and sub-groups of patients (Bergström et al., 2018, 2022a, 2022b, 2022c; Kiviniemi, 2014; Pirkola et al., 2009) have also failed to detect a significantly higher suicide rate in the Western Lapland catchment area as compared to other parts of Finland. This may suggest that those who have died by suicide in the Western Lapland region did not seek help in the first place, and thus remain undetected in studies including mainly those who have received mental health treatment. Since OD has about the same probability of suicide as adolescent mental health service users in other parts of Finland with lower annual suicide ratio as compared to Western Lapland, it remains possible that OD is improving outcomes for those receiving treatment relative to the those not receiving services in their local environment. To address these issues, there is a need for further studies with samples including all people who have died by suicide in the region.

The Western Lapland area has had low-threshold and immediate access to mental health services and also in this study had a relatively higher incidence of service users as compared to other psychiatric services in Finland. However, suicide-prevention may be challenging by the means of mental health treatment alone, as societal factors play an important role. It is important to note that the Western Lapland region has a more poverty as compared to many other regions of Finland. This may increase the relative population-level risk of suicides in the region of Western Lapland. Moreover, many young people from Western Lapland area are moving away from the region (THL, 2022) as job and education opportunities have been centralized to other parts of Finland due to a national trend towards urbanization. This together with an already small population base may significantly increase the relative annual proportion of those with a higher suicide-risk in the Western Lapland region, as those with better functioning and thus lower long-term risk for suicide are more likely to move away from the region (Bergström, 2020).

It should be noted that due the small population base of Western Lapland, the actual annual frequency of suicides in the region has been notably small in both national healthcare indicators and in this study sample, which significantly increases the risk of random variation. Nevertheless, this was a first nationwide longitudinal register-based comparison focusing on suicide rate and suicide risk-factors of all adolescents who received treatment under OD, and therefore results offered ecologically valid information on entire service-level 5-year suicide rate among the adolescent mental health service users. Our main findings aligned with earlier results concerning a sub-group of first-onset adolescent patients (Bergström et al., 2022a) and first-episode psychosis patients (Bergström et al., 2018).

### Strength and Limitations

The Finnish registers are considered to be a reliable source of information (Lahti & Penttilä, 2001; Sund, 2012). As they also enabled the inclusion of all persons in Finland who received adolescent psychiatric treatment within the pre-determined inclusion period, results offered ecologically valid information on suicide-mortality and suicide risk factors after the onset of adolescent mental health treatment in Finland. However, social and healthcare registers were not originally developed for research purposes and there may be inaccuracies. For example, some suicides may have been misidentified as accidents, or vice versa. We also missed some central variables known to associate with suicides, including socioeconomic status and prior suicide attempts.

There are some other limitations. First, due to the observational design and potential confounding by indication, we were not able to determine the causality between the certain treatment modalities (e.g. medication and hospital admissions) and suicides. Secondly, despite the large sample including all Finnish adolescent mental health patients within a pre-determined time-frame, the actual frequency of suicides remained small in both groups and especially in the OD-group, due to the small population base of Western Lapland region; there were only six suicides in the OD-group during the entire follow-up. Figure is aligned with our main conclusion, although low frequency increases the risk of random variation making it challenging to make firm statistical conclusions. Moreover, due to the small frequency of suicides under OD, we were not able to analyse whether there are OD-specific risk-factors associating with suicides.

Another limitation is that we lacked a reliable measurement of onset symptom severity. As diagnoses were not required and thus not always registered in OD, we were not able to ensure the comparability of groups. For example, based on previous studies (Bergström et al., 2022a), it is possible that in the OD group there is an overrepresentation of milder symptom cases due to the non-referral services. To ensure comparability of the two groups, we used population proportions in a comparison of overall 5-year suicide-ratio between OD and CG, which makes suicide-ratio (relative to population) between groups comparable even if there was an overrepresentation of milder symptom cases in OD. Even though primary outcome analysis was adjusted for potential confounders, there may still remain unidentified differences affecting suicidality.

## Conclusion

In a representative cohort of all adolescent mental health patients whose treatment was commenced in Open Dialogue based mental health services in the Western Lapland catchment area, there was no significant difference in 5-year suicide mortality as compared to mental healthcare commenced in other parts of Finland. This and previous findings suggest that the area’s relatively high overall suicide rate may be due to a subgroup of the local population at high risk for suicide who do not seek help or enter psychiatric services.

Findings also indicated that low-threshold services and dialogical mental health treatment alone may not prevent suicides more effectively than standard mental health care. As a result, there remains further need to study clinical and demographical characteristics of those who have died by suicide in the Western Lapland catchment area, including more detailed information on the psychiatric treatment that they have received or whether they have received treatment at all. In addition to the development of mental healthcare services, adequate suicide prevention may require further integration of different kind of services and focusing on regional and cultural factors, including social burden and poverty. The most significant risk-factor for suicide in the entire cohort of all adolescents who enrolled to the Finnish psychiatric services in 2003–2013 was (poly)substance abuse at the beginning of adolescent treatment: of those who died by suicide, one out of six had a diagnosis of substance abuse disorder (compared with 3 in 100 of those surviving). Substance abuse may be challenging to address via mental health services alone.

Findings of this and previous studies have consistently indicated that, as compared to standard services, the re-arrangement of mental health services based on the Open Dialogue approach is safe regarding overall suicide-risk, even though the OD-treatment is typically carried out on an outpatient basis and with a need-adapted medication strategy. However, apart from service type, the 5-year suicide ratio of an adolescent mental health service user’s was alarmingly high as compared to the general population of similar age. In the future, more information on suicides under OD-based services is needed to further develop these services ability to prevent suicides.

## Data Availability

The Health and Social Data Permit Authority Findata supervise the secondary use of all Finnish health and social care data including all of the data used in this study. Data can be accessed based on justified purposes. For more information and data permit applications see findata.fi.
